# Google Trend Analysis and Paradigm Shift of Online Education Platforms during the COVID-19 Pandemic

**DOI:** 10.3390/idr13020040

**Published:** 2021-05-12

**Authors:** Ashwani Kumar Kansal, Jyoti Gautam, Nalini Chintalapudi, Shivani Jain, Gopi Battineni

**Affiliations:** 1 Kasturba Institute of Technology, Abdul Kalam Technological University, Lucknow 226031, India; kansal.ashwani@gmail.com; 2 JSS Academy of Technical Education, Noida 201301, India; jyotig@jssaten.ac.in; 3 Telemedicine and Telepharmacy Centre, School of Medicinal and Health Products Sciences, University of Camerino, 62032 Camerino, Italy; gopi.battineni@unicam.it; 4 Department of Computer Science & Engineering, Indira Gandhi Delhi Technical University for Women, Delhi 110006, India; shivanijain13@gmail.com

**Keywords:** COVID-19, lockdown, digital learning, education systems, trend analysis

## Abstract

*Objective:* The largest pandemic in history, the COVID-19 pandemic, has been declared a doomsday globally. The second wave spreading worldwide has devastating consequences in every sector of life. Several measures to contain and curb its infection have forged significant challenges for the education community. With an estimated 1.6 billion learners, the closure of schools and other educational institutions has impacted more than 90% of students worldwide from the elementary to tertiary level. *Methods:* In a view to studying impacts on student’s fraternity, this article aims at addressing alternative ways of educating—more specifically, online education—through the analysis of Google trends for the past year. The study analyzed the platforms of online teaching and learning systems that have been enabling remote learning, thereby limiting the impact on the education system. Thorough text analysis is performed on an existing dataset from Kaggle to retrieve insight on the clustering of words that are more often looked at during this pandemic to find the general patterns of their occurrence. *Findings:* The results show that the coronavirus patients are the most trending patterns in word search clustering, with the education system being at the control and preventive measures to bring equilibrium in the system of education. There has been significant growth in online platforms in the last year. Existing assets of educational establishments have effectively converted conventional education into new-age online education with the help of virtual classes and other key online tools in this continually fluctuating scholastic setting. The effective usage of teaching tools such as Microsoft Teams, Zoom, Google Meet, and WebEx are the most used online platforms for the conduction of classes, and whiteboard software tools and learning apps such as Vedantu, Udemy, Byju’s, and Whitehat Junior have been big market players in the education system over the pandemic year, especially in India. *Conclusions:* The article helps to draw a holistic approach of ongoing online teaching-learning methods during the lockdown and also highlights changes that took place in the conventional education system amid the COVID pandemic to overcome the persisting disruption in academic activities and to ensure correct perception towards the online procedure as a normal course of action in the new educational system. To fill in the void of classroom learning and to minimize the virus spread over the last year, digital learning in various schools and colleges has been emphasized, leading to a significant increase in the usage of whiteboard software platforms.

## 1. Introduction

The impacts and effects of coronavirus disease in 2019 (COVID-19) are growing with unconditional boundaries and destination. It began in China, with prominent steps all over the world, though was not seen at first to be very scary or as devastating as it has become today with a more devastating second wave that has spread across the community at large. It has immensely affected human health as well as the economy and development of countries globally [1]. The action of the disease caused by the virus is unstoppable as it transmits via human-to-human interaction; non-immunity to this particular virus has fueled rapid transmission. It was declared a public health emergency of international concern by the World Health Organization (WHO) on 30 January 2020 [2]. However, as a preventive measure and to slow down the infection rate, various guidelines were issued by the WHO and respective nations that include lockdown, social distancing, quarantine, self-hygiene, isolation, working from home, and staying home, at a colossal economic developmental cost. The impact also directly falls on the education system, when all the schools and other educational institutions were shut for a couple of months. The United Nations Educational Scientific and Cultural Organization (UNESCO) reported that overall, 290 million students across 22 countries have been severely impacted by their education system [3]. In India, about 1.6 million school-going students and 2.6 million graduating students have been affected, with virtual education in place since the beginning of the pandemic [4]. The widespread proliferation of COVID-19 worldwide stimulated the need to amend and reassess the conventional mode of education. Educational experts and the government are focusing on digital education as a viable solution over conventional teaching methods. The UNO and several government policies prompted the beginning of online education for students globally.

With a second wave present for several months, there has been increasing use of teaching-learning through online mode because of the non-operation of schools, colleges and universities for an indefinite time and discontinuation of face-to-face teaching [5]. The COVID-19 pandemic has delivered us with an opening to flag the way for acquainting ourselves with digital learning [6]. The use of precise and applicable pedagogy with appropriate tools for online education depends on the proficiency of the teacher and student as well as the platforms used that include amalgamated communication and cooperation platforms. The most widely used are Microsoft Teams, Google Classroom, Zoom, and WebEx with the support of sharing contents in terms of video, documents, teaching materials, and others [7]. These also allow the tracking of student learning and assessment by using the assessment of various forms. Several whiteboard software and web conferencing tools that have gained attention during this crisis include Go Meet, Realtime Board, ezTalks Meeting, Zoom, AWW app, Web Whiteboard, Whiteboard Fox, Microsoft conferencing, Cisco WebEx Meeting, Google Hangout meets, Microsoft Teams, join.me, Cisco Jabber, iMeet, Electromeet, Lifesize, Fuze, Adobe connect and GoToMeeting, etc. These platforms have been used by educators to provide classroom learning on an online mode of teaching. Most whiteboard software tools are freely available with limited features; some have premium versions (paid version) with all the features set. The educator chooses their software as per the requirements, such as the number of students, number of hosts, working hours, data transfer, and time duration. Apart from college and school teaching, many educational platforms provide tuition and extraneous courses such as CueMath, Vedic Math, English speaking courses, Simplilearn teaches cybersecurity, Cloud, DevOps, Artificial Intelligence and Data Science, and many other online courses to provide valuable education.

In this article, text mining is performed to analyses the most trending patterns of words and phrases during the pandemic, keeping education prospects as the focus. Moreover, data on internet search, social media [8], and Google analysis [9] have been used to mine information regarding the topic and analyze the leading aspects in terms of word search patterns and online platforms used throughout the pandemic. In this work, the authors have aimed to seek trend analysis on online platforms preferably used in the Indian education system. The authors have explored the Google trend on different learning platforms such as Zoom, WebEx, Google Meet, Microsoft, etc. The trend of using the top-rated online education platforms, namely Byju’s, Veduantu, Whitehat Junior, and Khan Academy for school students and Unacademic, Udemy, Syamam and Edx for UG and PG level courses were considered. Analysis and inspection of the Google trends of last year (15 February 2020 to 15 February 2021) on the different online platforms are presented to show how online platforms have grown due to the pandemic and its social and economic impact in the country. The increase in Google trends explicitly symbolizes the perception and work initiated, delivered and commissioned in various parts of the world [10,11].

This study majorly focused on the adoption and validation of various online educational platforms and presents the inevitability of various psychological distresses that are causing additional health damage and adverse life satisfaction among students. A comprehensive review of different virtual classroom platforms has been performed based on Google trend analytics to put across factors, outside of epidemic symptoms, that have impacted the lives of students during the COVID-19 pandemic. The authors have also analyzed a dataset called the COVID-19 Open Research Dataset (CORD-19), which is a resource of more than 400,000 scholarly articles, and examine the text patterns and psychological effects after understanding the student mental conditions. User analysis of different platforms has been performed to identify the popularity of individual learning platforms, and it uncovered a far-ranging consequence with the reduction in the mental wellbeing of students due to the current pandemic.

## 2. Literature Study

The article mainly has two sections that are coincidentally related to each other. While analyzing educational system changes and the shift of paradigm towards online teaching, the trends globally make a lot of sense to acquaint with the ongoing pandemic situations worldwide. The model of text mining seems to have a greater impact in seeking insights from the various sources of data and correlating them with existing trends using Google trend patterns. Google Trends is an online social networking searching tool that includes real-time and non-real-time data from the local users. This allows us to measure interest in a particular topic across search, from around the globe, right down to city-level geography. Google Trends data are available in real-time; increasingly, it is helping people around the world to explore the global reaction to major events. It is anonymized (no one is personally identified), categorized (determining the topic for a search query) and aggregated (grouped). Data scientists analyze text using advanced data science techniques. The data from the text reveals customer sentiments toward subjects or unearths other insights from sources such as customer reviews, assembling valuable insights. There are various techniques for text mining, analyzing and retrieving insights from text [12]. Recently, social reviews and post have been used to study the impact of the COVID-19 outbreak [13]. Similarly, general public opinion has also been identified using trend analysis [14]. In the educational system, a comprehensive study has been carried out to ensemble various interdisciplinary approaches to teaching and learning [15]. The phase shifts from various levels of the education system such as the primary, secondary and tertiary system are evaluated using trend analysis on various socio-economic backgrounds [16]. Google trend data has been analyzed to generate a pattern of consequences due to the COVID-19 impact using data science and mining techniques [17,18]. Seeking information for emerging trends in the usage of online learning apps and whiteboard software tools, Byju’s teaching and learning system has been used to identify breakthroughs and challenges that an education system would come across while implementing the novel approach of learning [19]. During the adverse situation of the pandemic, how universities have come up with their process of teaching, learning, assessment and an approach that would fit for the teachers as well as students in an online system of education [20]. There has been a complete change of convention, shifting the focus to remote access and monitoring as well as pedagogy. A comprehensive review has mention the discontinuation of face-to-face learning to adapt to a new system of learning during COVID days as well as near-future demands for the same approach [21]. A report highlights the loss of international mobility, loss of schools setting preparedness towards the new paradigm and impact on the vocational education system in a global scenario [22]. On a similar note, a study from the Indian perspective gives a way forward for opting for technology-driven approaches and digitization of content for preparedness towards such a crisis. In the current context of a pandemic through the use of information and communication technologies (ICT), emotions and motivations are managed through meaningful learning among students [23]. The Indian context is also studied through student populations to derive a resilient education system for the near future [24,25].

## 3. Materials and Methods

This article aims to produce significant insights from textual mining from the dataset that is available online and to validate this with trends through Google trend analysis on various software packages and tools that are being used by the teaching-learning fraternity.

### 3.1. Google Trend Analysis on Whiteboard Software’s

Using virtual classrooms or Whiteboard software (Miro, SF, USA), the educator teaches the mass population whilst maintaining social distancing. This software provides real-time interaction with students with no geographical barriers. The main difference between the white-board software and the video or web conferencing is that in web conferencing many people connect via the Internet to share ideas or talk to each other as a team, whereas the white-board software is similar to classroom teaching. To gain insights from the search pattern, the authors have used the R tool to seek patterns that incorporate the present education system through whiteboard software and its growth in the education system in India. We have used the gtrends R package to identify the Google trends for online education. In this research, we have used three different parameters; first, we have searched the terms to those companies who are providing the platforms for online education. The main contributor in these areas is ZOOM, Cisco Web-Ex, Microsoft Teams, and Google Meet. Next, we have compared the online platform providing the school education concepts for the school level students. Here, we have compared the top online platforms, such as Byju’s, Vedantu, Whitehat Junior and Khan Academy.

### 3.2. Text Analysis

This analysis was conducted in a two-step process. Primarily, we applied text mining on COVID-19 text data of 2020 to find the different patterns. The COVID-19 dataset was extracted from Kaggle, which is an open research platform [26]. Text pre-processing is performed on the dataset. After the basic pre-processing and curation of textual data, the document term matrix (DTM) is constructed. As the DTM size is very large, the sparsity of the documents is removed and the final DTM is created. Once the DTM has been constructed, the term frequency-inverse document frequency (TF-IDF) is applied and the 310 most occurring words related to COVID-19 are extracted. To identify similar words from the textual data, hierarchical clustering is performed. With hierarchical clustering, similar, related words are identified and are plotted in the form of dendrogram representation, which is a tree-like structure in which branches at the same level represent similar words.

In the second step, we have used Google trends to identify how online education is promoted during the pandemic time. The R statistical application has been incorporated to identify the progress of different online platforms in India and their impact on the Indian education system. We identified a few search queries that assisted us to execute Google analysis. For related queries, we have used the package gtrendsR, Reshape2 and tidy verse package to show these queries.

Query: <Zoom meeting app download, Zoom Chinese app>,
<Google meet for pc, how to use Google to meet>,
< Byjus bnat>,
<Whitehat junior salary>,
< Whitehat junior dashboard>,
< Khan academy>,
< Un academy scholarship test>,
< Udemy free courses during a lockdown, Swayam biomedical research>.

The query mentioned above has been segregated to create a correlation between the trends that Google Trend showed along with the common terms used while searching for the leading institutional online courses and tools that were used during the period of a pandemic. Various APIs used through the R package is the internal core algorithms that are put in place for carrying out the lead based on the objective of the article.

## 4. Results

The outcome of research activities mentioned in the section above has been interpreted as the patterns that are sought through the dataset using text mining as a predictor of concern as well as the trends towards usage of online platforms for a new paradigm of teaching and learning system.

### 4.1. Pattern Identification on COVID-19

**Figure 1:**
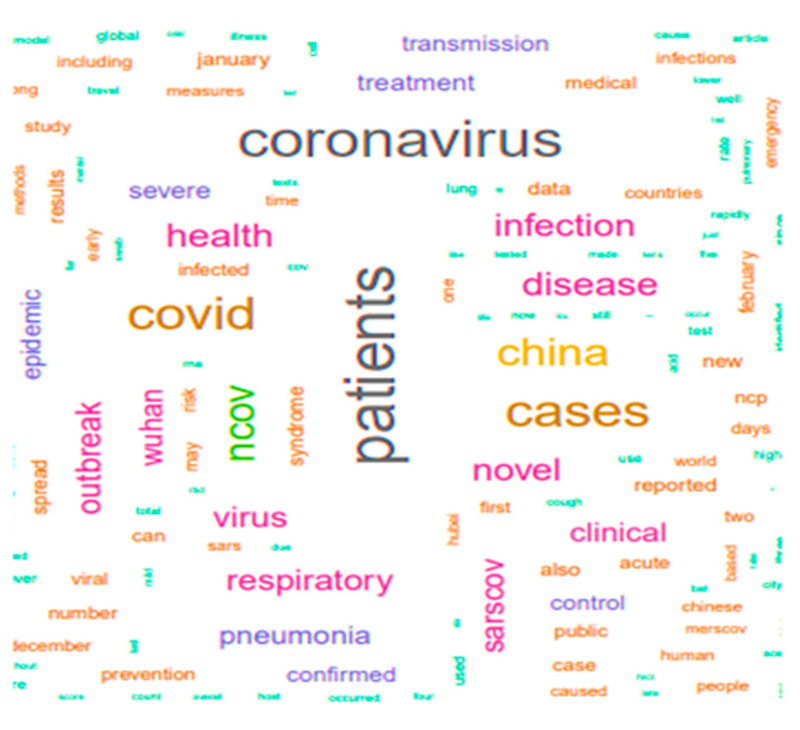
Word cloud of most common search patterns.

Figure 1 represents the word cloud of common terms that appeared during the COVID-19 pandemic. The dataset mining was performed to identify these words having a higher impact on the social being; however, the education system during this emergency has been seen to be a less prominent sentiment. Figure 1 depicts the presentation of high-frequency search terms in the word cloud diagram. More often, coronavirus patients have more prevalent search patterns along with health care, various syndromes and China seen.

**Figure 2:**
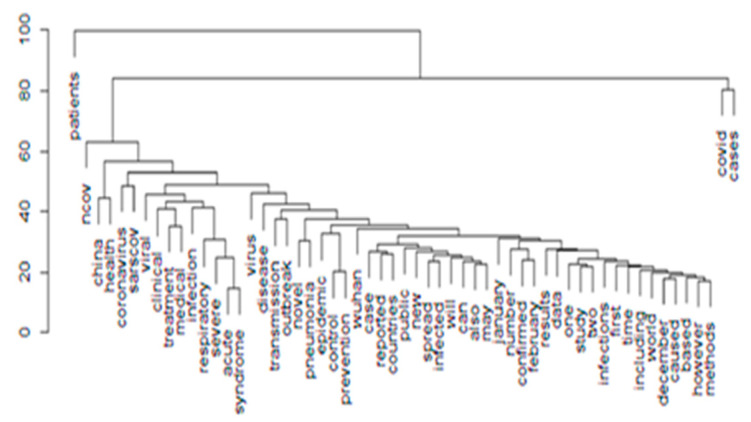
Cluster dendrogram.

On a similar note, while clustering the hierarchy of the words, Figure 2, a dendrogram representation arranges significant patterns in the tree-like structure. Here, China was related to words such as infection and diseases and occurred below words coronavirus and SARS-CoV, a virus that infers that china is the initiating location. The impact on the education system is least seen in the diagram as words related to it are far below the level of hierarchy represented in the figure.

The patterns of COVID-19 as represented in both the figures highlight the consequences rather than the cause of the infection, which signals the resultant effects on the education system, wherein people are scared of continuing their education, the closure of institutions and shortage of in-person teaching-learning methodologies.

### 4.2. Google Trend Analysis

**Figure 3:**
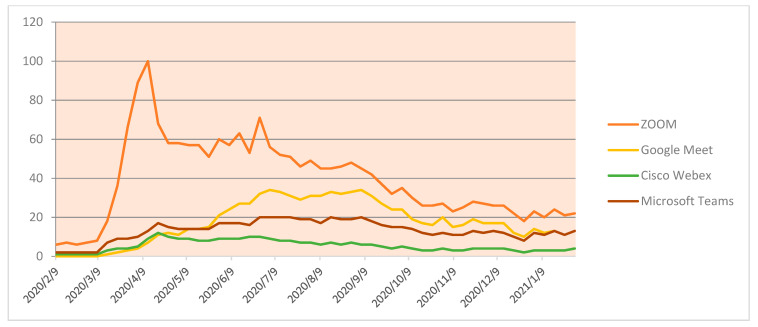
Comparison of different online platforms.

While analyzing the trends and patterns of Google searches throughout the pandemic period, various online platforms have been incorporated by educators and learners to continue with the ongoing process of the education system. Figure 3 represents a comparison of the usage of online platforms. It clearly shows that there are regular hits on such legitimate search keywords with particular names of the platforms which were making its footfalls on the new education system. It is evident that during the lockdown the Zoom application has been widely used and reached a peak on 12 April 2020. As of now, Zoom has 200 million users in India. Zoom has achieved popularity due to its easy setup, white-board facility, virtual background and 100% recording ability.

**Figure 4:**
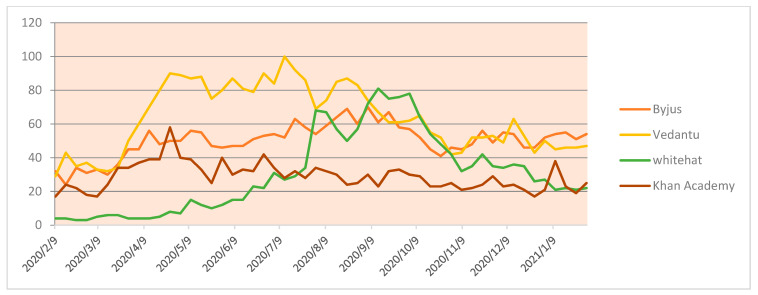
School-level online platforms.

On the other hand, when learning platforms with an additional mentor and tutor facilities are looked at, Byju’s gained importance among the other players in the market. During the lockdown, Vedantu and Khan Academy also performed quite a remarkable job in promoting learning among university-going students, as represented in Figure 4. Whitehat Junior is also the most popular online application for school-going kids. They have invested manpower, resources and extended financial implication towards marketing. As seen from the trends, Whitehat Junior is on extensive marketing during May and June, thus seeing a huge hit on Google trends after that period. Swayam is a government initiation to educate UG- and PG-level course, showing growth in this area also and Udemy fee courses are the most searched on Google. Udemy has seen massive growth in the last year; their turnover in the year 2020 was $100 million.

**Figure 5:**
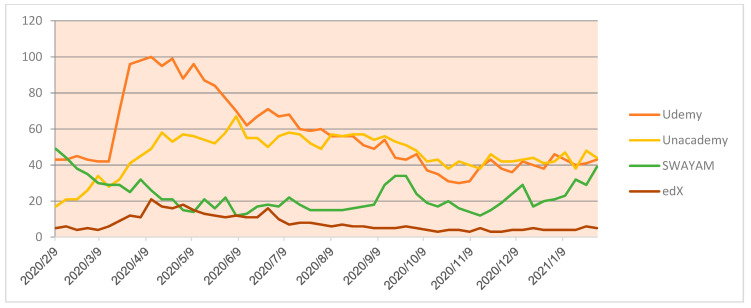
UG and PG course provider.

Additionally, the comparison of the online platforms that are providing education at UG- and PG-level courses reveals different notes on exhibiting various courses at the disposal of potential learners. While using keywords such as Udemy, Unacedamy, Swayam and Ed-X, Figure 5 highlights the number of hits as per the corresponding dates.

## 5. Discussion

Researchers and scientists have always been way ahead in taking on challenges and bearing the torch of a bright and prosperous future as far as the COVID-19 struggle is concerned. To channelize their potential and bear on them to develop solutions by joining hands together in this pandemic time, the government has also been supportive of various initiatives and funding. Online education is promoted in India in the form of web lectures, assignments, presentations, and online classes in this catastrophic situation of lockdown. The Indian government has taken all the major steps to ensure that every child should receive an education. That apart, the government released a fund of 15 million for different research plans in cure and disease prevention along with synchronizing current distress of the education system. Here, we have summarized the impact on online education in India during this pandemic period.

### 5.1. The Socio-Economic Impact through the New Education System of Online Learning

Whenever such a pandemic with a health crisis happens, the biggest loss occurs on the economic front. However, the losses incurred by society in terms of the educational perspective are also not to be sidelined. With the advent of the online education system being an alternative to the conventional education system, the primary cause of economic drain is the purchase of packages including high-speed internet to abide by the changes in the learning paradigm. However, it was used as a preventive measure taken to prevent the spread of COVID-19 but became a prime reason for the use of modern technologies in India at the user’s end to a community level. The trend result from this research also supports greater usage of technologies, which is a significant change for the educational system in India. It is also projected to take a lead to a manifold increase in ed-tech markets with a sharp rise in many organizations. The players in the market such as Byju’s, upGrad, Vedantu, CLEducate, Imarticus Learning, Topper, Swayam, Udemy, Simplilearn, and many more are performing at their maximum extent to cater for the needs during the pandemic [6]. The regular classes for the undergraduate and postgraduate students are significantly seen from the Google trends as the result of having compulsory education based on digital education, and authorities have also been supportive in this regard. To, conclude, on the adaptation of a new education system, there will be a significant contribution in multiple ways towards economic development, development of a modern and technological education system, developing capacities for economic development for individuals with an increase in employment and promoting novel ideas of entrepreneurship. With the advent of the online education system as a preventive measure taken to prevent the spread of COVID-19, it can become a prime reason for sustenance for modern and technological India. This kind of impact not only spread across India; the consequences are also far-reaching to the entire globe [27].

### 5.2. Social Impact of Digital Learning

As per the result, it is significant that more than 90% of students are dependent on the online platforms for continuing their education, thus a primary concern of COVID spread would be decreased. Having remote learning, maintaining social distancing and taking video lectures has the largest impact on Indian students. According to evidence, some school students and parent are in fear, while some are enjoying the new way of learning. However, primary kids are enjoying as the educator send the assignment in the form of animated videos. The approaches of online education through various whiteboard software packages in the urban areas as well as the rural community are significantly seen with the little hurdle of internet connection in the rural areas. But excessive digital learning has affected students and educators with several mental health issues by increasing the stress level. Several students are stressed due to closure of lack of access to resources provided in schools and colleges. Students belonging to sub-urban and rural areas do not have internet facilities to gain knowledge through a digital medium. Children with special education needs such as Autism disorder are facing challenges [28]. With its varied impact globally, digital learning has become a new way of learning to foster the education system through an assortment of technology and resources together [29].

### 5.3. Recommendations and Future Scope

From the study of real-time data from Google trends and existing dataset analysis, it is however unjustified to infer that whatever is happening in the education system is good for the sake of current and next-generation nation builders. On analyzing text from the open dataset, it has been proved that current trends lie with coronavirus and during this phase of crisis education system is changed to online modes as depicted by the Google trends. Through a holistic approach that has been incorporated in data collection, querying and analysis, the article lacks certain section which can be taken up by the future researchers. Before jumping onto the limitations, the authors would like to put forward certain recommendations regarding the implementation of full flagged online accommodation of the education system in India. One of the major concerns is the availability of reliable power supply and pervasive connectivity of the Internet thus delivering stress. There is a lack of internet connection facilities with unreliable power supplies in suburban and rural areas of India which interferes with the expulsion of the online education system. Along with this, students of all age groups are more adhesive to an online gaming console, social media which becomes a prime reason for distraction among students and leading to a lack of enthusiasm for online learning. A comprehensive awareness drive is recommended to create a concise view on the change that took place because of the COVID pandemic and we have to live with it. Also, there is a paucity of the proper atmosphere as online learning had never been promoted or given prime importance in the traditional educational system. Due to all these challenges, Educators and the Indian government are facing a lot of confrontations in implementing the Online education system [30]. But some measures can be incorporated such as digital videos in online learning, implementing virtual reality, improvisation of communication through social media channels, e-mails, student chat groups and many others. The limitation of this article is non-generalizability, which can be taken up as a research area with massive online/offline survey-based analysis of actuals that the teaching-learning fraternity has to say. A comprehensive meta-analysis of psychological, pedagogical and technological blends in the online learning system can be another area of work that can be taken up [31].

## 6. Conclusions

As hypothesized in the beginning, as a result of this research, it can be concluded that a significant paradigm shift has occurred in the education system post-COVID-19 outbreak. Digital learning is a new way of learning in India as well as worldwide. As Google Trend suggests, it may change the learning method of the country. Government authorities and educators need a lot of effort to make it a success. Acceptance of life with the COVID-19 pandemic has been established through text mining but a great learning opportunity with digital means in the education domain for students and as well as educators, which would be the new inception of the modern and technological education system in India. However, in a rural area, it is a burden and as the resources are limited in the current situation to apply it as a fully-fledged system, it will take a longer time. Amid technological advancement [32] in data representation, analysis and assessments, by incorporating additional measures, an online education system will become a prime resource to the administrators of the education system in India and globally.

## Data Availability

Not applicable.
